# Influence of Acquired and Genetic Risk Factors on the Prevention, Management, and Treatment of Thromboembolic Disease

**DOI:** 10.1155/2014/859726

**Published:** 2014-06-26

**Authors:** Raghid Kreidy

**Affiliations:** Department of Vascular Surgery, Saint George Hospital, University Medical Center, University of Balamand, Youssef Sursock Street, P.O. Box 166378, Achrafieh, Beirut 1100 2807, Lebanon

## Abstract

Prevention, management, and treatment of venous thromboembolism requires understanding of the epidemiology and associated risk factors, particularly in recognizing populations warranting prophylaxis, in evaluating patients with high risk situations, and in determining the duration of anticoagulation required to minimize recurrent thrombosis and to avoid postthrombotic syndrome. The present paper reviews recent advances concerning acquired and genetic risk factors for venous thrombosis, analyses individual risks related to age, and focuses on thrombotic genetic risk factors and the synergistic gene-environment and gene-gene interactions and their importance in the management and treatment of venous thromboembolic disease.

## 1. Introduction

Lower extremity deep vein thrombosis (DVT) remains a common and serious medical condition manifesting in patients with recognized or unrecognized risk factor or complicating the outcome of critical ill and surgical patients. When it is misdiagnosed or improperly treated, DVT may lead to pulmonary embolism, the most devastating complication of acute DVT. Pulmonary embolism is responsible for 10% of death among hospitalized patients and is the most common preventable cause of death in these patients. American Heart Association statistics document two million cases of DVT each year with the incidence of DVT increasing as the population ages [1]. Pulmonary embolism (PE) accounts for 200 000 deaths each year and the annual cost of the treatment is measured in billions of dollars [2]. Postthrombotic syndrome is also a costly and morbid long term complication of DVT with severe adverse socioeconomic impacts [3].

The appropriate management of venous thrombosis requires a thorough knowledge of diagnostic and treatment modalities. However, an understanding of the underlying epidemiology and associated risk factors is equally essential. Once risk factors for venous thrombosis are recognized, appropriate management and treatment may ensue. Promptly diagnosed and properly treated, lower extremity DVT may become a benign disease [4].

The purpose of this review paper is to determine the influence of acquired and genetic risk factors on the prevention, management, and treatment of venous thromboembolic disease and on reducing the incidence of PE, recurrent venous thrombosis, and postthrombotic syndrome. Age-related risk factors, interaction between acquired and genetic risk factors, and the effect of multiple and complex prothrombotic genetic defects on venous thromboembolism will be particularly discussed.

## 2. Discussion

The development of clinically manifest venous thrombosis most often occurs with the convergence of multiple acquired and genetic risk factors. These factors vary according to ethnic and geographic distribution of the populations [5]. The simultaneous presence of multiple risk factors is a prerequisite for thrombosis, with synergistic gene-gene and gene-environment interactions often increasing the risk above the sum of individual risk factors [6].

Lower extremity deep venous thrombosis (DVT) is predominantly a disease of older age [7]. The incidence of DVT increases exponentially with age for both idiopathic and secondary DVT rising nearly 90-fold between 15 and 80 years of age with a relative risk of 1.9 for each 10-year increase in age [5, 8]. DVT is uncommon in young adults and very rare before the age of 20 years [5].

A recent study demonstrated that inherited thrombophilia is the most common risk factor for venous thrombosis among patients younger than 50 years with prevalence of at least threefold increase comparing to the control group [9, 10]. Thrombophilia should be screened in patients under the age of 50 even with the presence of a transient risk factor. Young adults present usually with severe forms of venous thrombosis. They should be assessed for acquired and particularly for genetic factors. This permits to extend the duration of anticoagulant therapy in high risk patients reducing the incidence of postthrombotic syndrome and venous thromboembolic recurrence. Pregnancy and treatment with oral contraceptives or estrogen drugs especially when associated with inherited thrombophilia represent a frequent cause of DVT among young female patients [9, 10]. Screening for thrombophilia among young females with strong family history of venous thromboembolism (VTE) before starting estrogen therapy is warranted. Congenital abnormalities of the inferior vena cava (IVC), essentially when associated with inherited thrombophilia, are an underdiagnosed cause of venous thrombosis [11]. They are responsible for 14% of iliac vein thrombosis among young adults and should be excluded in young patients with spontaneous iliofemoral venous thrombosis, especially when recurrent venous or thrombosis resistance to anticoagulants is observed. For IVC congenital anomalies without thrombophilia, at least 6-month treatment with anti-vitamin K is required. Long term and sometimes life-long oral anticoagulation are recommended if IVC congenital abnormalities are associated with severe thrombophilia or with recurrent DVT [12].

Elderly patients tend to have all the usual risk factors associated with VTE but also face additional risk conferred from a higher incidence of comorbidities, immobility, and the hypercoagulability associated with aging. In the elderly patient, clinical diagnosis can represent a challenge because clinical symptoms and signs are subtle, atypical, nonsensitive, and nonspecific. Advanced age is an independent risk factor for VTE [13] and has been reported in some series to be the most frequent risk factor for VTE [14]. Hospitalization, immobility, hip fracture, heart failure, and chronic renal failure increase considerably the risk for venous thrombosis in the geriatric age group [15, 16]. Unless contraindicated, every elderly patient, bedridden or having multiple comorbid conditions, admitted for sepsis, cancer, critical medical condition, or surgery, must receive thromboprophylaxis with dose adjustment according to creatinin clearance if required. Early ambulation, strict control of heart failure, and chronic renal failure and aggressive treatment of hip fractures are highly recommended for these geriatric patients [16].

Obesity has been associated with increased thrombotic risk, particularly in patients admitted for acute medical illness, trauma, or surgery [17]. Mechanical prophylaxis and thromboprophylaxis with adjusted dose according to increased bodyweight must be considered in this high risk group.

The risks for VTE increase essentially when surgery is performed in patients with advanced age, under general anesthesia, for trauma, cancer, and major orthopedic disabling diseases [18]. Thromboprophylaxis should be extended for five weeks postoperatively in patients undergoing major orthopedic surgery or abdominopelvic surgery for cancer [19].

The incidence of recurrent DVT is higher among patients less than 65 years of age and patients with idiopathic DVT, irreversible thrombotic risk, primary hypercoagulopathy essentially factor V R 506 Q-Leiden (FVL) mutation, and hyperhomocysteinemia [20].

In a recent published study, the authors detected FVL mutation among 25.7% of all patients with recurrent DVT and among 66.6% of patients with recurrent DVT younger than 60 years [14]. Prolonged duration of oral anticoagulation is suggested when patients carriers for FVL mutation develop iliofemoral venous thrombosis or severe postthrombotic syndrome.

Oger et al. suggested that varicose veins are an independent risk factor for DVT only among women and those greater than 65 years of age [21]. In a previous published study, the author did not observe a significant difference in the incidence of DVT related to varicose veins with gender [14]. However, 77% of the patients reported with varicose veins were above 65 years of age, emphasizing the importance of thromboprophylaxis in this particular risk group [14].

Venous thrombosis following immobilization and bed rest is frequently bilateral. In chronically immobilized persons residing at home or at a nursing home with no other risk factor for VTE, recent guidelines do not suggest routine thromboprophylaxis [19].

Venous thrombosis increases with malignant tumors and increases much more with metastasis, with chemotherapy, and particularly with thrombophilia. However, in outpatients with cancer and indwelling central venous catheters and with no additional risk factors for VTE, recent recommendations do not suggest routine prophylaxis with low molecular weight heparin [19].

Air travel is associated with a threefold higher risk for VTE with an 18% higher risk for each 2-hour increase in travel duration [22]. This risk increases significantly when additive known risk factors including severe obesity, advanced age, limited mobility, previous VTE, recent surgery or trauma, active malignancy, pregnancy, estrogen use, or known thrombophilic disorder are reported. For high risk patients, hydration, avoiding drinking alcohol, frequent ambulation, calf muscle exercise, sitting in an aisle seat if feasible, wearing graduated compression stockings, putting comfortable clothes, and using anticoagulants in some selected cases are highly suggested [19].

A fine balance exists between anticoagulant, procoagulant, and fibrinolytic factors. A hypercoagulation state or thrombophilia is common and is observed among 50% to 70% of patients with unexplained VTE [23], 39.5% to 53.5% of pregnant women [24, 25], and 72% of individuals with travel-related VTE [26]. In some countries with high prevalence of prothrombotic genetic polymorphism, thrombophilia was reported to be the second most common cause of VTE [14]. Recent studies suggested that thrombophilia not only predisposes to the development of venous thrombosis but also seems to interfere in the development of postthrombotic syndrome either directly by prolonging residual venous thrombosis or indirectly by increasing venous thrombotic recurrence rate [27, 28]. Assessment for genetic risk factors for VTE allows to avoid thrombogenic treatment (estroprogestative treatment) and to prevent venous thrombosis in high risk conditions (pregnancy, trauma, surgery, and long-haul air travel) [29]. Oral anticoagulation should be extended for prolonged periods among patients with severe thrombophilia, which ultimately reduces venous recurrence rate and postthrombotic syndrome [29].

Among the inherited thrombophilias, factor V-Leiden gene mutation is the most common predisposing factor, accounting for 10% to 20% of VTE in large population studies [30]. The prevalence rate of factor V-Leiden in the general population varies from 0% to 15% according to ethnicity [5, 31]. The allele mutation is low in African, Asian, and South European populations (1%–3%) and high in Mediterranean populations (13.6% in Syria and 13.4% in Greece) [32]. Lebanon exhibits one of the highest frequencies of FVL mutation in the eastern Mediterranean and in the world with a prevalence of 14.4% in the general population [33]. The prevalence of FVL mutation is particularly increased when venous thrombosis occurs in children, young patients, pregnant patients, patients with family history of VTE, and patients with spontaneous, extended DVT resistant to anticoagulation [14, 34]. FVL mutation should be screened in this high risk subgroup of patients. FVL mutation interacts with other concurrent acquired and thrombophilic conditions such as cancer, oral contraceptive use, hormonal replacement therapy, pregnancy, surgery, long-haul air travel, and associated prothrombotic genetic abnormalities and essentially prothrombin G 20210A mutation to increase the risk of incident venous thrombosis [35]. The relative risk for venous thrombosis is calculated to be 2- to 3-fold for the prothrombin mutation alone and 20-fold for a combination with FVL mutation [36]. In a recent study, the author reported three young adult patients harboring this combination [36]. Two of them, carriers for an additive MTHFR mutation with increased homocysteine level, presenting for bilateral extensive lower extremity venous thrombosis and severe PE, were extremely resistant to anticoagulant therapy and required IVC filter insertion, aggressive, and prolonged anticoagulation. FVL acts as a concurrent risk factor in individuals with other prothrombotic polymorphisms and hyperhomocysteinemia leading to a synergistic gene-gene interaction increasing the potential risk for venous thrombosis [37]. The duration of anticoagulant therapy among patients carriers for FVL mutation depends on the nature and extension of the thrombosis, the underlying risk factors, the presence of a previous VTE, and the presence and severity of concurrent prothrombotic genetic mutation [36]. Patients with homozygote mutation for FVL and patients with combined heterozygote mutation for FVL and prothrombin are considered for extended long term treatment with anticoagulant therapy [38].

Increased homocysteine plasmatic level is associated with both arterial and venous thrombosis and results essentially from MTHFR C 677 T and MTHFR A 1298 C mutation. These mutations may or may not lead to hyperhomocysteinemia, depending on the homozygosity or heterozygosity of the mutation, coinheritance with another mutation, or the presence of concurrent B vitamin deficiency [39]. The association between MTHFR C 677 T and MTHFR A 1298 C genetic polymorphism and the increased risk for VTE is still controversial [40, 41]. Ray et al. suggest that the risk of these mutations is weak and is increasing in older patients and with coinheritance with other mutations [42]. Akar et al. confirmed that MTHFR A 1298 C is an independent risk factor for venous thrombosis and that MTHFR C 677 T and A 1298 C mutation are associated with 3- to 5-fold increased risk for thrombosis [43]. Our findings suggest that MTHFR C 677 T and MTHFR A 1298 C mutation either alone or associated with other prothrombotic genetic defects essentially FVL increase the risk for venous thrombosis [14].

Factor V H 1299 R polymorphism has been reported to be a possible risk factor for the development of VTE with a high prevalence in the world (9.5–1 5%) [44]. We suggest to screen understudied polymorphism such as MTHFR A 1298 C and factor V H 1299 R in severe extended cases of VTE resistant to anticoagulation and not explained by the most usual mutations [45].

## 3. Conclusion

Recognizing acquired and genetic risk factors allows for better prevention, management, and treatment of venous thromboembolic disease. Risk stratification is important in determining which patient requires prophylaxis in high risk situations, in counseling patients related to their risk associated with contraception, pregnancy, and hormonal replacement, and in understanding who requires further consideration because of young or advanced age, malignancy, and recent surgery. The number of acquired factors predisposing to thrombosis usually outweighs the number of prothrombotic genetic factors. Few thromboses are generally caused by inherited thrombophilia alone. The finding that patients with thrombophilia can harbor more than one prothrombotic state have further increased the relevance of the congenital thrombophilic states. The association of two or more prothrombotic genetic defects to FVL mutation increases considerably the risks and the severity of venous thrombosis.

Thrombophilia screening should be tailored to accommodate a population's risk factor. In countries with high prevalence of prothrombotic genetic polymorphism, thrombophilia should be tested among patients younger than 50 years even with a transient risk factor and patients with conditions highly suggestive of hypercoagulation state. This permits to stratify individual patients in to high and low risk for incident and recurrent venous thromboembolism, targeting prophylaxis to those who would benefit most, extending the duration of anticoagulation therapy in high risk patients, and, ultimately, reducing the occurrence of recurrent venous thromboembolism and postthrombotic syndrome.
